# Practicability of the Serum-Therapy in the Treatment of Hog-cholera1Read before the joint-meeting of the Iowa State Veterinary Association and the Iowa State Agricultural Society, January 10, 1899.

**Published:** 1899-03

**Authors:** W. B. Niles

**Affiliations:** Ames, Iowa


### PRACTICABILITY OF THE SERUM–THERAPY IN THE TREATMENT OF HOG–CHOLERA.[1]

By W. B. Niles, D.V.M.,
AMES, IOWA.

Our Secretary has assigned me a subject which I cannot discuss as satisfactorily as I would like, for the reason that the use of serum in the treatment of hog-cholera has not been tried sufficiently to determine whether it is practical or not.

Knowing, however, the great interest veterinarians and stock-owners have in this subject, I consented to give you freely the benefit of what information I could gather relative to the practicability of what is now generally called the serum treatment for cholera in swine.

As the preparation of the serum and the way in which it is supposed to produce immunity and cure disease have been thoroughly described in two papers read before this body by Dr. Peters, of Nebraska, I will not touch upon these points, but at once proceed to the practical side of the question.

Any line of treatment for disease, whether directed toward prevention or cure, to be practical must possess two qualities: first, it must succeed, and, secondly, the treatment must be of such a nature that it can quite generally be put into practice.

If a given treatment will not cure a considerable percentage of diseased animals or prevent the contraction of the disease, it is not practical, no matter how easily applied or how inexpensive. On the other hand, if a given line of treatment cures every case and will render all or a large percentage immune, it is not practical unless so inexpensive and of such a nature that it can come into general use. Measured in this way, we say that the practicability of the

[1] Read before the joint-meeting of the Iowa State Veterinary Association and the Iowa State Agricultural Society, January 10, 1899.

serum treatment of hog-cholera depends, first, upon whether this line of treatment will render hogs immune against the disease and cure the sick; and, secondly, whether the serum can be obtained and used at a figure which the swine-raiser can afford to pay.

Taking these questions up in order: Will the serum cure the sick and render well hogs immune? This is a hard question to answer at the present state of our knowledge, and I am no better prepared to answer it than some others. I do not consider it necessary for the treatment to do both in order to be practical, providing it possesses the second qualification. If it will do but one, if it will cure a large percentage of the sick ones, or prevent the disease when given to the well, either before or after exposure, it may be considered a practical line of treatment. If it will cure the disease it should also produce immunity if given before exposure; but it may prevent the disease and still not cure the sick.

During the past season serum from several sources has been used in different parts of the country. Early in the summer the energetic pharmaceutical firm of Parke, Davis & Co. sent a man into this State for the purpose of trying the effects of their serum. Dr. E. A. A. Grange, who made the experiments, informed me that the results were not good, and that this serum consequently was not put upon the market. The Southern Vaccine Company, of Galveston, Texas, has for some time been experimenting with a cholera-serum, and now have a preparation to distribute to farmers and veterinarians for practical tests. So far the value of their serum is not known. In Nebraska, as you are aware, the Experimental Station has for three years been trying a serum prepared by Dr. Peters, that I believe has given varying results. I understand that a report from that station will soon be out, giving the results of some late experiments which, according to my information, have not been so satisfactory as was hoped, especially in the way of preventing the disease.

Dr. Peters kindly informed me that during 1898 only the curative power of the serum had been tested, for previous experiments taught that its preventive quality was not very great. If, when given before exposure, it will not prevent the disease after subsequent exposure, it seems to me doubtful that it can be very successful as a cure. We regret to hear that in Dr. Peters's hands it has failed as a preventive, for most of us have placed our faith in its preventive action.

As is also well known, the Department of Agriculture, Bureau of Animal Industry, last year used considerable serum in Page

County, and have this year continued the work on a larger scale. In an address at Omaha, last September, Dr. Salmon, Chief of the Bureau, stated, in regard to the results obtained last year, that of about two hundred and fifty animals in infected herds, over 75 per cent. were saved by the serum treatment. He also said that this year (up to September) the results had been better, indicating that 80 per cent. of the animals in infected herds could be saved.

I regret to say that in the limited number of experiments I have recently made for the Bureau, the results have not been so good. I have, however, only dealt with herds where the disease was very virulent and had gained a good foothold before the serum was used, and I presume I did not have the strongest serum. It seems to me that it is not reasonable to suppose that any treatment will cure those that are badly diseased; and from what I have learned from different sources there is nothing yet within reach that will do this.

While we may remain unable to cure the sick, especially those showing well-marked symptoms, I believe, however, that a serum of sufficient antitoxic power can be obtained to render, at least, a certain percentage of swine immune. As to what percentage ought to be saved in this way, I am not prepared to say. The Bureau has a *Bulletin* in print giving some results of serum work. This will no doubt give us much information on the serum treatment.

Granting that it will be so successful as to warrant its use, will it be practical to use it? Can it be obtained in sufficient quantities, and will the price admit of its use?

One prominent veterinarian thinks that even if successful the treatment will likely have little practical value on account of the necessarily limited amount that can be produced. He states that in his State it would have taken something like four hundred and fifty barrels to treat all the hogs lost in 1896.

I believe he is unwarranted in his conclusions, for the reason that all our swine need not and will not be treated. It must not be supposed, and we must not make the mistake of thinking, that the serum treatment, even if successful, is all that we need to "help us out of the woods" in dealing with this cholera question. It should be used systematically in connection with efficient sanitary regulations. If only used spasmodically here and there, by a farmer or a neighborhood, the spread of the disease will not be arrested, the loss not much reduced, and little good will be done. Like tuberculin, anthrax-vaccine, blackleg-vaccine, and other like preparations, it can best be used under professional supervision. Dr. Salmon states that it should form a valuable addition to the resources of the State in eradicating the disease, and we can readily see that when in early spring cholera appears in a neighborhood, if the neighboring herds could be rendered immune, it would very much assist in stamping out the outbreak. If used systematically in this way by the State authorities in all parts of the State when hog-cholera appears, but a comparatively small percentage of the swine kept would need the serum treatment.

The price at which serum will be furnished cannot yet be definitely determined. The Southern Anthrax Vaccine Company state that the price will be about ten cents per dose. This, even in additions to the veterinarian's fee for using it, would not make the treatment expensive. Dr. Peters thinks it can be produced for from ten to fifteen cents at a good profit.

Since observing the numerous Marshall County outbreaks the past season, I am more than ever impressed with the fact that we must have a quarantine system in order to deal satisfactorily with this disease.

I want to emphasize the fact that the serum treatment should be used in connection with quarantine. It is certainly easier to stamp out the disease in one herd than to treat all the hogs of a township with serum or by any other method.

In some townships the disease began in early spring in a single herd, then spread over almost all the township by extending along the highway from farm to farm. In early spring there were probably not more than six or eight such farms in the county. Why may we not suppose that if these had been rigidly quarantined and the herds destroyed, the loss would have been very light? Like other things, there is often a time when it may be "nipped in the bud."

Without systematic efforts in other directions the serum treatment, no matter how successful, will not serve to control the disease.

As swineraisers we must do our part in carrying out sanitary rules and regulations, and we ought to insist that the State assist us by looking after those who persist in undoing all the good work we may do by letting their herds run at large, and in other ways encouraging the spread of the disease.

Trusting that a serum of sufficient antitoxic power will yet be produced which will very much aid in the suppression of the disease, I leave the question with you for discussion.

---

Dr. C. L. Megowan fills the post of city food-inspector for Sacramento, Cal.

